# Sex differences in energy intake, sweat rate, and electrolyte loss among world-class archers during competition

**DOI:** 10.1080/15502783.2025.2528532

**Published:** 2025-07-03

**Authors:** Ozcan Esen, Stuart Goodall

**Affiliations:** a Northumbria University, Department of Sport, Exercise & Rehabilitation, Faculty of Health and Life Sciences, Newcastle upon Tyne, UK; b North-West University, Physical Activity, Sport and Recreation Research Focus Area, Faculty of Health Sciences, Potchefstroom, South Africa

**Keywords:** Archery, carbohydrate, hydration, sports nutrition

## Abstract

**Background:**

Limited evidence reports energy and macronutrient intake during competitive archery and whether any sex differences exist. Understanding these factors will provide insights into physiological demands and could inform more effective strategies to optimize performance for all archers in this precision-demanding sport. This study aimed to evaluate sweat rate (SR), sweat electrolyte loss, and energy intake (EI) in world-class archers throughout competition.

**Method:**

Eight (4 females) elite-standard archers (age: 21 ± 2 and 19 ± 1 years; body mass: 65.1 ± 2.8 and 60.1 ± 4.1 kg; stature: 179.3 ± 5.1 and 162.3 ± 0.8 cm, for males and females, respectively) from the Turkish National Archery Team participated. Data were collected over four days; assessments of hydration, SR, sweat composition ([Na^+^] and [K^+^]), and nutritional intake were completed at the same time each day.

**Results:**

Male archers consumed more total energy (2,889 vs. 2,353 kcal, *p* = 0.007) and carbohydrates (5.3 vs. 3.9 g/kg, *p* = 0.046) compared to females, with intake fluctuating based on competition demands (i.e. match duration, and the total distance walked). SR and sweat [K^+^] loss, were greater in males compared to females (*p* ≤0.006). In contrast, no sex differences were observed in other hydration parameters (sweat [Na^+^] loss, urine-specific gravity). Moreover, all archers maintained adequate hydration status throughout the competition, with no differences in pre- and post-competition hydration levels (*p* > 0.05).

**Conclusion:**

This study expands on previous research by incorporating sex-specific analyses, demonstrating that while energy and carbohydrate intake varies between male and female archers, hydration-related variables remain consistent.

## 1. Introduction

Recurve archery, featured in the Olympic Games, demands intermittent and repetitive shooting where achieving precision is crucial for success. While it is widely recognized as a mentally demanding sport due to the psychological stressors associated with competition, such as anxiety, tension, and pressure, archery also places significant physical demands on athletes, requiring fine motor control, upper body strength, and endurance [1,2]. The physical strain involved in archery, as evidenced by the force needed to pull the bowstring (i.e. ~19 kg) [3], demonstrates the physiological requirements of archery. While physiological demands of archery are distinct from those of endurance or high-intensity sports, the activity still requires considerable energy expenditure (EE) as the predicted daily EE has been reported up to ~4000 kcal/d in world-class male archers [4]. However, there remains limited knowledge about the physiological aspects of archery, and critical areas remain underexplored, particularly regarding female archers and key factors such as sweat rate, electrolyte loss, and macronutrient intake during matches. Addressing these gaps in the literature will provide valuable insights into the physiological demands of archery and contribute to the development of more tailored nutritional and hydration strategies for athletes, thereby supporting enhanced performance.

To date, only one study has reported energy and macronutrient intake during an archery tournament. In that study, world-class male archers’ daily energy and carbohydrate intake ranged from 2,563 to 3,986 kcal/d and 4.0 to 7.1 g/kg BM/d, respectively [4]. The authors suggested that the repetitive, intermittent nature of the sport, coupled with its extended duration, suggests that energy requirements may vary significantly across competition phases. This may also be particularly relevant for female archers, whose physiological responses to exercise and nutritional needs may differ from their male counterparts due to hormonal and metabolic differences [5,6]. Furthermore, habitual energy and absolute CHO intake might be influenced by the effects of the menstrual cycle phase on appetite regulation, gastrointestinal symptoms, and food cravings (e.g. sweet foods) [7,8], which may affect real-world fueling. Additionally, to our knowledge, no investigation has reported macronutrient and fluid intake during competitive archery performance for either sex. Importantly, an understanding of these factors may aid the design of nutritional strategies to optimize performance.

Research into other precision-based sports has recently emphasized the importance of hydration and nutrition on performance, with findings suggesting that slight dehydration can impair muscular endurance, postural control, and fine motor skills [9]; factors all crucial for archery. Increased sweat rate and electrolyte loss can lead to a reduction in brain plasma volume and disruptions in cognitive processing and brain function [10]. These changes are also associated with negative effects on mood, vigilance, and alertness [11,12]. Together, these impairments can compromise an archer’s fine motor control, decision-making, and precision. In a study of world-class male archers, mean 24-h urine-specific gravity (USG) results indicated euhydration throughout a tournament [4]. However, no studies have yet examined the sweat rate and electrolyte losses (i.e. sodium [Na^+^] and potassium [K^+^]) in archers during competition, leaving a gap in our understanding of how these variables have the capacity to affect performance. Given that archery competitions often last for several hours, and involve repeated bouts of physical exertion, it is plausible that sweat rate (SR) and electrolyte imbalances could accumulate over time, potentially impacting archery performance, especially when competitions are held in hot and/or humid conditions. Investigating these factors is critical for developing optimal hydration strategies tailored to the unique demands of archery

Thus, the present study aimed to address these gaps by evaluating SR, electrolyte loss, and energy intake (EI) in world-class male and female archers during competition. Understanding these factors will provide essential insights into the physiological demands of archery and could inform more effective hydration and nutrition strategies to optimize performance for both male and female athletes in this highly precision-demanding sport.

## 2. Materials and methods

### 2.1. Participants

Eight (F = 4, *M* = 4) elite-standard archers from the Turkish National Archery Team participated in the current study. Archers included tier 5 (e.g. Olympian and Olympic or World or European Championship medalists, *n* = 4 [F = 1, *M* = 3]) and tier 4 athletes (e.g. international athletes, *n* = 4 [F = 3, *M* = 1]) [13]. All archers trained an average of 12 × 2.5 hours archery-based and 6 × 1 h strength and conditioning-based training sessions weekly at the Turkish Archery Federation Performance Centre. Before the study, participants provided written informed consent and completed a health screening questionnaire. The research protocol received ethical approval from the Institutional Review Board of Northumbria University (Reference no: 4243).

The physical profiles of the participants were (mean ± SD, males, females and total average, respectively): age, 21 ± 2, 19 ± 1 and 20 ± 2 years; body mass, 65.1 ± 2.8, 60.1 ± 4.1 and 63.7 ± 4.1 kg; stature, 179.3 ± 5.1, 162.3 ± 0.8 and 170.8 ± 9.2 cm; body fat percentage, 8.7 ± 2, 26 ± 3 and 16 ± 10 %; and maximal oxygen uptake (O_2max_), 47.9 ± 2.7, 40.9 ± 2.9 and 44.3 ± 4.6 mL·kg^−1^·min^−1^. While O_2max_ was assessed using the graded treadmill protocol [14] following 12 h fasted state, body fat percentage was estimated using the eight-site skinfold method [15]. Details regarding hormonal contraceptives or other medication use were not obtained, however, female archers performed during the luteal phase, which was determined by individual declaration of the 20th day from the beginning of menses.

### 2.2. Study design

The study involved data collection over four days during an Archery World Cup Stage 1 in May (2023). The daily competition schedule varied (see Table 1) and took place on an outdoor grass pitch; environmental conditions of ambient temperature, humidity, pressure, and wind speed were: 23.7 ± 2.1ºC, 50 ± 14.4%, 1,009 ± 2 mbar, and 1.4 ± 3.6 mph, respectively. Athletes’ diets during the tournament were nutritionist-led.

**Table 1.** Match load variables and predicted daily total energy expenditure (TEE) (representative of average daily data from archers).

		Day 1	Day 2	Day 3	Day 4
Arrow no	*Overall*	178 ± 3	123 ± 28	101 ± 29	97 ± 43
	*Male*	177 ± 3	125 ± 24	113 ± 28	100 ± 35
	*Female*	179 ± 1	121 ± 28	90 ± 25	94 ± 50
Duration (min)	*Overall*	119 ± 6	88 ± 29	57 ± 21	53 ± 24
	*Male*	119 ± 8	89 ± 9	67 ± 21	56 ± 21
	*Female*	119 ± 2	96 ± 38	47 ± 17	49 ± 26
Walking distance (m)	*Overall*	5119 ± 169	3698 ± 1004	2538 ± 945	2260 ± 1007
	*Male*	4970 ± 70	3553 ± 409	2975 ± 909	2333 ± 817
	*Female*	5268 ± 91	3844 ± 1344	2100 ± 760	2188 ± 26
Predicted TEE (kcal)	*Overall*	3131 ± 296	2987 ± 302	2844 ± 345	2262 ± 246
	*Male*	3406 ± 82	3218 ± 130	3157 ± 169	2493 ± 105
	*Female*	2856 ± 128	2756 ± 242	2532 ± 120	2031 ± 57

### 2.3. Quantification of competition load

The competition and training/warm-up sessions were observed and documented. Due to competition regulations, archers were not permitted to use electronic or computerized monitoring devices during the events. The variables selected for analysis from the competition data included the number of arrows shot, match duration, and the total distance walked, which are defined elsewhere [4]. Daily predicted total energy expenditure (TEE) was estimated using a validated equation the resting metabolic rate [16] and the metabolic equivalent of tasks during competition [17]. A body mass-based predictive equation validated in athletes of a similar standard (aged 18–35 years) [18] was used to estimate resting metabolic rate.

### 2.4. Hydration assessment

Hydration status was determined by obtaining urine samples from participants after waking up, pre- (~30 min before), and post-competition (~15 min after), and before (~30 min before) bedtime. Samples were subsequently analyzed using a digital refractometer (Atago 3730 Pen-Pro Dip-Style Digital Refractometer, Washington, USA) to determine USG as an indication of hydration status throughout the day. The participants’ hydration levels were categorized as euhydrated (USG < 1.020), minimally hypo-hydrated (USG 1.020–1.024), or hypo-hydrated (USG > 1.024), based on previously established criteria [19].

### 2.5. Body mass and fluid balance

Pre- and post-session body mass (BM) was recorded using a digital scale (Seca 769, Seca GmbH, Hamburg, Germany) with participants wearing minimal clothing. Participants were instructed to void their bladders before both measurements. Any urine passed during the sessions was collected in pre-weighed containers and weighed afterward. Fluid intake and food items were ad libitum, with access to water and sports drinks, and energy bars/gels, provided during the sessions. All fluid and food items were weighed before being consumed, and any remaining fluid or food was weighed again after consumption. Total sweat loss was estimated by calculating the difference between pre- and post-session BM, adjusting for fluid and food intake, and urine output. Minor changes due to metabolic water loss or substrate oxidation were considered negligible [20].

### 2.6. Sweat collection and assessment of sweat [Na^+^] and [K^+^]

To assess sweat [Na^+^] and [K^+^] concentrations, sweat was collected using absorbent patches (Tegaderm + Pad, 3 M, St. Paul, MN, USA) placed at two skin sites: the scapula and the thigh. Before applying the patches, the skin was cleaned using deionized water and sterile gauze. Patches were affixed to the skin for the entire session and removed immediately afterward. The sweat was extracted from the patch via a syringe method and analyzed on-site according to a previous study [21]. Sweat [Na^+^] and [K^+^] levels were measured using an ion-selective electrode device (Horiba Na-11, K-11, respectively, Kyoto, Japan) immediately after the competition (~15 min). Total Na^+^ and K^+^ loss was estimated by multiplying sweat [Na+] and [K^+^] by the total sweat volume [22].

### 2.7. Nutritional intake

Dietary intake was documented using food diaries, supplemented with photographs to enhance portion size accuracy and ensure completeness of records. Participants who were already experienced in using food diaries received a brief refresher from the lead researcher on how to accurately record their intake the day before the competition. This combined method of using food diaries and photographic records is both effective [23] and reliable [24] for assessing EI in free-living athletes. Meals (breakfast, lunch, and dinner) were provided at the athletes’ accommodation in buffet style and designed by a certified sports nutritionist following the Athlete’s Plate Nutrition Educational Tool to ensure balanced nutritional composition without specifying exact macronutrient or calorie information [25]. Additionally, the team nutritionist worked with the athletes throughout the season, set up a fueling station at the competition site to provide specific nutritional support. This included carbohydrate-rich products (e.g. sports drinks [~36 g of CHO for 500 ml drink], gels [~25 g of CHO for 50 g gel], granola [~18 g of CHO for 35 g pack]), protein sources (e.g. protein bars [~20 g of protein for 55 g bar], nuts, powders [~25 g of protein for 30 g]), and ergogenic aids (e.g. caffeine [~200 mg of caffeine per 100 ml shot], inorganic nitrate [~6 mmol of nitrate per 70 cl of shot]), based on established sport nutrition guidelines [19]. Participants were free to consume food and fluids ad libitum, with no obligation to finish meals or snacks. All drink containers and food items were weighed before and after consumption to accurately measure intake. Dietary intake data were analyzed using Nutritics software (Nutritics Ltd, Ireland).

### 2.8. Statistical analysis

All data are presented as mean ± SD with normality assessed using the Shapiro – Wilk test. Descriptive statistics only are provided for aspects of competition load (see Table 1). A two-way mixed-design ANOVA was employed to determine differences both within subjects (time-points) and between subjects (sex) in daily and in-competition energy, macronutrient, fluid intake, and hydration status (i.e. USG in 24-h and between days, sweat rate, sweat Na^+^, and K^+^) across the study period. When a significant sex × time-points interaction or main effect of time-points was found for any parameters, Bonferroni corrected paired sample t-tests were performed to determine specific differences. Effect sizes were calculated as Partial eta square (*η*_*p*_^2^), which varies from moderate (≥0.07) to a large effect (≥0.14) [26]. Linear regression analysis using Pearson’s coefficient (*r*) was used to understand associations between TEE vs. EI and CHO intake. Statistical significance was set at *p* < 0.05 for all tests and data were analyzed using SPSS (v28, IBM Corp., Armonk, NY, USA) or Excel ([regressions], Microsoft, Washington, USA).

## 3. Results

**Figure 1.**
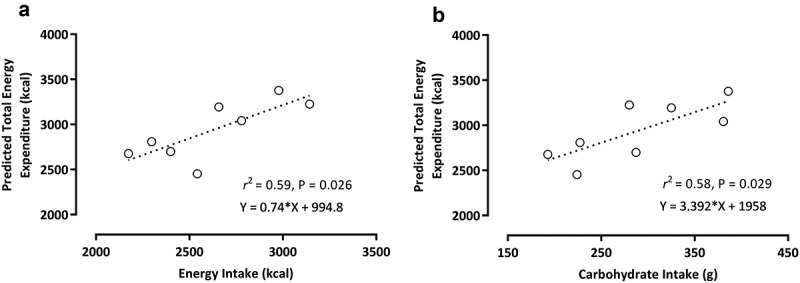
Linear regression analysis between the predicted total energy expenditure and average energy intake (a) and average absolute carbohydrate intake (b).

All variables presented a normal distribution according to the Shapiro – Wilk test (*p ≤* 0.05). Daily absolute (*F* = 26.18; *p* < 0.001; *ŋ*_*p*_^2^ = 0.81) and relative EI (*F* = 26.18; *p* < 0.001; *ŋ*_*p*_^2^ = 0.81) differed across the 4-days (see Table 2). Absolute EI was higher on day 1 than on days 3 (*p* = 0.004), and 4 (*p* < 0.001), but not day 2 (*p* = 0.395). Additionally, EI were higher on day 2 than day 4 (*p* < 0.011); and higher on day 3 than day 4 (*p* = 0.001). There was a difference in mean EI for sex (male > female, 2,889 kcal *vs*. 2,353 kcal, respectively, *p* = 0.007). There was also an EI*Sex interaction (*F* = 4.91; *p* = 0.012; *ŋ*_*p*_^2^ = 0.45). Post-hoc analyses indicated that absolute EI was higher in males than females on days 1 (*p* < 0.001) and 2 (*p* = 0.029), but not on days 3 and 4 (both *p* > 0.05). Relative EI was higher on day 1 compared to days 3 (*p* = 0.007) and 4 (*p* < 0.001), but not on day 2 (*p* > 0.05). Relative EI was also higher in days 2 (*p* = 0.01) and 3 (*p* = 0.001) compared to day 4. There was no difference in relative EI between days 2 and 3 (*p* > 0.005). There was also an EI*Sex interaction effect (*p* = 0.042). Post-hoc analysis demonstrates that relative EI was higher in male compared to female archers on day 1 (*p* = 0.16), but not on days 2 and 3 (*p* > 0.05). There was a positive relationship between average EI and TEE (*r*^2^ = 0.59, *r* = 0.77, *p* = 0.026) (Figure 1a).

**Table 2.** Daily relative and absolute energy (EI_relative_, EI_absolute_), carbohydrate (CHO_relative_, CHO_absolute_) and protein (PRO_relative_, PRO_absolute_), intake, daily fluid intake (Fluid_daily)_ and hourly CHO intake during competition (CHO_competition_) over the 4-day tournament. Recommended daily CHO intake range (3–7 g/kg BM/day) is based on standard sports nutrition guidelines [14].

		Day 1	Day 2	Day 3	Day 4
EI_absolute_ (kcal)	Overall	3041 ± 556^cd^	2745 ± 457^d^	2606 ± 297^d^	2094 ± 259
	Male	3526 ± 238*	3069 ± 353*	2720 ± 393	2243 ± 279
	Female	2557 ± 192	2421 ± 288	2492 ± 125	1946 ± 141
EI_relative_ (kcal/kg/day)	Overall	48.5 ± 4.6^cd^	43.9 ± 6.7^d^	41.8 ± 4.9^d^	33.5 ± 3.7
	Male	54.2 ± 4.2*	47.1 ± 4.7	41.8 ± 5.6	34.5 ± 4
	Female	42.9 ± 5.4	40.7 ± 7.5	41.8 ± 5	32.5 ± 3.6
CHO_absolute_ (g)	Overall	330 ± 90^bcd^	290 ± 111^cd^	306 ± 60^d^	188 ± 88
	Male	371 ± 109	340 ± 129	346 ± 28	239 ± 97
	Female	289 ± 52	241 ± 75	265 ± 56	136 ± 39
CHO_relative_ (g/kg BM)	Overall	5.7 ± 1.1^d^	4.7 ± 1.8	4.8 ± 0.9^d^	3.2 ± 1.2
	Male	6.5 ± 0.8	5.3 ± 2.1	5.3 ± 0.7	4.1 ± 0.9
	Female	4.8 ± 0.5	4.1 ± 1.3	4.4 ± 0.9	2.6. ± 0.7
CHO_competition_ (g/gh)	Overall	43 ± 1.3^bd^	27.3 ± 8.5^d^	44.2 ± 1.9^bd^	13.8 ± 0.4
	Male	43 ± 1.7	27.3 ± 12.5	43 ± 2	14 ± 0.3
	Female	42.5 ± 1	27.5 ± 8.5	45.2 ± 1	13.7 ± 0.3
PRO_absolute_ (g)	Overall	167 ± 33^d^	154 ± 36	166 ± 34^d^	127 ± 28
	Male	166 ± 40	168 ± 39	177 ± 34	132 ± 39
	Female	169 ± 30	140 ± 32	155 ± 34	122 ± 28
PRO_relative_ (g/kg BM)	Overall	2.8 ± 0.5	2.5 ± 0.6	2.7 ± 0.5^d^	2.1 ± 0.3
	Male	2.9 ± 0.6	2.6 ± 0.8	2.7 ± 0.5	2.2 ± 0.3
	Female	2.7 ± 0.5	2.3 ± 0.6	2.6 ± 0.6	2.0 ± 0.2
Fluid_daily_(L/day)	Overall	3.8 ± 0.2^bd^	3.6 ± 0.2^d^	3.9 ± 0.2^bd^	3.4 ± 0.2
	Male	4 ± 0.1	3.7 ± 0.2	4 ± 0.1	3.5 ± 0.1
	Female	3.7 ± 0.2	3.5 ± 0.2	3.7 ± 0.2	3.2 ± 02

bcd: day 1 is significantly higher than day 2, 3 and 4; cd: day 2 is significantly higher than day 2 and 4; c: day 1 is significantly higher than day 3; d: day 1, 2 or/and 3 is significantly higher than day 4; *: males > females.

Daily absolute (*F* = 11.02; *p* < 0.001; *ŋ*_*p*_^2^ = 0.65) and relative CHO intake (*F* = 11.26; *p* < 0.001; *ŋ*_*p*_^2^ = 0.62) were different across the 4-days (see Table 2). Absolute CHO intake was higher on days 1 (*p* = 0.001), 2 (*p* = 0.29), and 3 (*p* = 0.01) compared to day 4, but there was no difference between days 1, 2 and 3 (all *p* > 0.05). There was no effect of sex or a Sex*time interaction (both *p* > 0.05). Relative CHO intake was higher on day 1 (*p* < 0.001) and day 3 (*p* = 0.001) compared to day 4, but there was no difference between days 1, 2, and 3; and days 2 and 4 (all *p* > 0.05). There was a difference in mean relative CHO intake for sex (male > female, 5.3 g/kg *vs*. 3.9 g/kg, respectively, *p* = 0.046), but no sex*time interaction (both *p* > 0.05). There was a positive relationship between average absolute CHO intake and TEE (*r*^2^ = 0.58, *r* = 0.76, *p* = 0.029) (Figure 1b).

Daily absolute (*F* = 5.35; *p* = 0.008; *ŋ*_*p*_^2^ = 0.47) and relative PRO intake (*F* = 5.52; *p* = 0.007; *ŋ*_*p*_^2^ = 0.48) were different across the 4-days. Absolute PRO intake was higher on days 1 (*p* = 0.05) and 3 (*p* = 0.01) compared to days 4, but there was no difference between days 1, 2, and 3 (all *p* > 0.05). There was no effect of sex or a sex*time interaction (both *p* > 0.05). Relative PRO intake was higher on day 3 compared to day 4 (*p* = 0.044), but there was no difference between days 1, 2, and 3; and days 2 and 4 (all *p* > 0.05). There was no effect of sex or a sex*time interaction (both *p* > 0.05).

CHO intake during the competition differed across the 4-days (*F* = 71.95; *p* < 0.001; *ŋ*_*p*_^2^ = 0.92) with no difference between sexes (*p* > 0.05). On days 1 and 3, CHO intake during the competition was higher than on days 2 (*p* = 0.015; 0.009, respectively) and 4 (both *p* < 0.001). Additionally, CHO intake during the competition was higher on day 2 than on day 4 (*p* = 0.05). Daily fluid intake differed across 4-days (*F* = 80.80; *p* < 0.001; *ŋ*_*p*_^2^ = 0.93) with no difference between sexes (*p* > 0.05). On day 1 and 3, daily fluid intake was higher than on days 2 (*p* = 0.013; 0.009, respectively) and 4 (both *p* < 0.001). On day 2, fluid intake was higher than on day 4 (*p =* 0.037). Average fluid intake was 0.66 ± 0.21 L/h during the competition with no difference between sexes (*p* > 0.05). Average inorganic nitrate intake (via beetroot juice supplementation) and caffeine intake before the competition were 7.5 ± 1 mmol/d and 3.2 ± 0.2 mg/kg, respectively, with no difference between sexes (*p* > 0.05).

**Figure 2.**
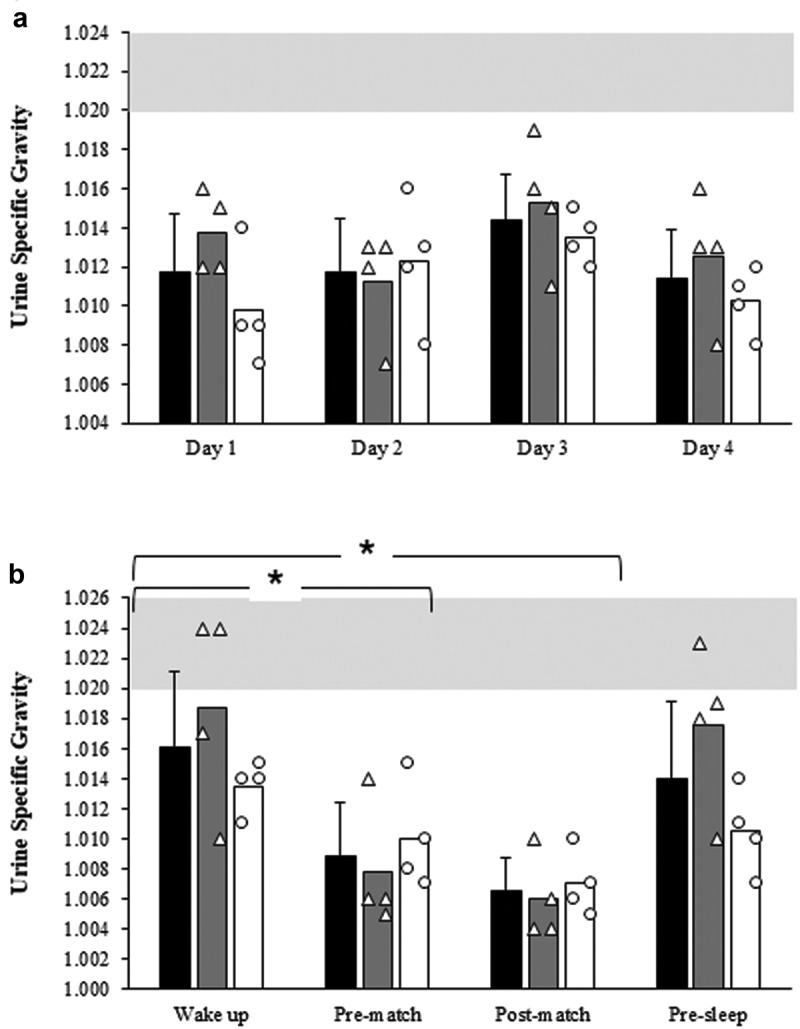
Urine specific gravity (USG) values for 24-h across 4 days (2a) and at different time points during a day (2b). *indicates significant differences between days and time-points (*p* < 0.05). White triangles: individual male values; white circles: individual female values; black bars: mean value of overall; gray bars: mean value of males; white bars: mean value of females gray shadow: minimal threshold that dehydration starts (USG values above 1.020 indicate the onset of dehydration).

USG results for 24-h were not different across the days (*p* > 0.05, Figure 2a). There was no difference in USG results across days between sexes (*p* > 0.05) but there was a difference in USG across time-points throughout the day (*F* = 13.89; *p* < 0.001; *ŋ*_*p*_^2^ = 0.69, Figure 2b). Specifically, USG was higher after waking up than pre- (*p* = 0.029) and post-competition (*p* = 0.028). There was no difference in USG results across time-points during a day between sexes (*p* > 0.05).

**Figure 3.**
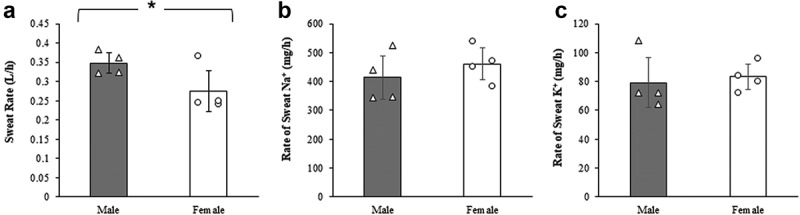
Sweat rates (L/h), and rate of sweat sodium [Na^+^] and potassium [K^+^] (mg/h). White triangles: individual male values; white circles: individual female values; gray bars: mean value of males; white bars: mean value of females. *indicates significant differences between sexes.

SR, BM change pre- and post-competition, body sweat [Na^+^] and [K^+^] concentration, and the rate of sweat Na^+^ and K^+^ loss for all archers can be seen in Table 3. SW, and the rate of sweat Na^+^ and K^+^ loss is illustrated in Figure 3. When comparing sex, the sweat rate was greater in male than female archers (*p* = 0.002). Sweat [Na^+^] did not differ between sexes when expressed either as mmol/L or mg/h (both *p* > 0.05). Sweat [K^+^] was greater in male than female archers when expressed as mmol/L (*p* = 0.006), but not as mg/h (*p* > 0.05).

**Table 3.** Whole body sweating rate, body mass change, whole body sweat sodium [Na^+^] and potassium [K^+^] concentration, and the rate of sweat Na^+^ and K^+^ loss by sex.

	Overall	Male	Female
Pre-event Body Mass (kg)	63.7 ± 4.1	64.2 ± 3.2	63.1 ± 4.7
Post-event Body Mass (kg)	63.3 ± 4.1	63.7 ± 3.2	62.9 ± 4.7
Body Mass Change (%)	0.58 ± 0.32	0.75 ± 0.19	0.33 ± 0.50
Sweat [Na^+^] loss (mmol/L)	45.2 ± 9.7	40.2 ± 11.7	50.2 ± 6.7
Sweat [K^+^] loss (mmol/L)	3.8 ± 1	3 ± 0.5	4.6 ± 0.6^#^

*males > females, ^#^females > males.

## 4. Discussion

This is the first study that has assessed sex differences in energy and macronutrient intake, and markers of hydration (i.e. SR, sweat [Na^+^], and [K^+^] losses) among world-class archers during a tournament-based competition. The main findings indicate that males consumed higher total energy and carbohydrates compared to females, with intake fluctuating based on competition demands (e.g. number of arrows shot, duration, and walking distance). These fluctuations suggest a periodized nutrition strategy that aligns with the demands of high-volume competition days, while also emphasizing the need to tailor carbohydrate intake based on individual athlete requirements to ensure adequate fueling throughout the tournament. Concerning hydration, SR and sweat [K^+^] loss was greater in male compared to female archers. In contrast, no sex difference was observed in other hydration parameters (i.e. sweat [Na^+^] loss, USG). Moreover, all archers maintained adequate hydration status throughout the competition, with no differences in pre- and post-competition hydration levels. This study expands on previous research by incorporating sex-specific analyses, demonstrating that while energy and carbohydrate intake varies between male and female archers, hydration-related variables remain consistent.

### 4.1. Daily energy and macronutrient intake

In line with previous research [4], the daily EI of both male and female archers in this study matched their predicted TEE. The average EI across the 4 days was positively associated with TEE (Figure 1a) and the regression equation allows us to determine for every 1 kcal of EI consumed, there would be a 0.74 kcal increase in TEE. Likewise, carbohydrate intake differed across the competition days, with the highest absolute and relative intakes observed on days 1 and 3 (see Table 2). The average absolute CHO intake across the 4 days was positively associated with TEE (Figure 1b) and the regression equation allows us to determine for every 1 g of CHO consumed, there was an associated 3.39 kcal increase in TEE. Male archers consumed an average of 5.3 g/kg BM of carbohydrate (ranged 6.5–4.1 g/kg BM), while females averaged 3.9 g/kg BM (ranged 4.8–2.6 g/kg BM). Although the amounts met the recommended CHO intake for low-to-moderate intensity athletes (3–7 g/kg BM) [19], the differences were likely due to higher energy needs linked to body size and magnitude of muscle mass [19]. However, all archers modulated CHO intake similarly across the tournament. These findings support the consideration of individualized nutritional approaches to ensure archers meet their carbohydrate requirements, particularly on high-demand days [27].

### 4.2. Carbohydrate and fluid intake during competition

The results show that CHO intake during the competition itself followed a similar trend, with higher intakes on days 1 and 3 (~43 g/h) compared to days 2 and 4 (~20 g/h), which is within the recommended intake (30–60 g/h) for exercise lasting between 1.5 and 3 h [19,28]. Previous research has highlighted the importance of carbohydrate availability in maintaining cognitive function and precision during extended competition by maintaining substrate delivery for the brain resulting in preventing brain function due to potential hypoglycemia, which is crucial in sports like archery that demands sustained focus and fine motor control [29]. The increased CHO intake observed on these days likely reflects the archers’ strategic efforts to maintain physical performance. The average fluid intake of 0.66 ± 0.21 L/h aligns with general hydration guidelines for athletes, which typically recommend 0.4–0.8 L/h of fluid intake depending on factors such as temperature, sweat rate, and exercise intensity [30]. Notably, there was no difference in fluid intake between sexes, suggesting that both male and female archers maintained similar hydration practices during competition. Inorganic nitrate and caffeine supplementation were at an average of ~7.5 mmol/d and ~3.2 mg/kg before the competition, respectively, both of which fall within the recommended range for enhancing athletic performance [19]. Given the efficacy of these substances also depends on timing, to ensure ergogenic benefit, supplementation was administered according to established guidelines for optimal pre-competition timing (~1 and 3 h before competition for caffeine and inorganic nitrate, respectively). While adherence to timing protocols was high, minor deviations occurred due to individual schedules. Even small timing shifts can affect outcomes, as caffeine’s effects vary with circadian rhythms [31] and nitrate responsiveness fluctuates within individuals across days [32]. Previous research has reported that nitrate [33] and caffeine supplementation [34] could positively influence cognitive functions such as reaction time, concentration, and executive function during physical exertion. Taken together, it may reflect an awareness of the potential performance-enhancing effects of these compounds in competition settings by world-class archers.

### 4.3. Hydration

The results of the current study regarding USG provide valuable insights into the hydration status of elite archers across multiple days of competition. USG results for 24-h did not show significant variation across the days or between sexes. USG results were also <1.020 at time-points during the day although it was highest after waking-up. These findings expand on the only previous study [4] by supporting that both male and female archers maintained euhydration status during the day and throughout the event. Male archers had a higher SR than females, consistent with studies linking greater SR in males to higher BM and metabolic heat production [35,36]. In terms of electrolyte loss, sweat [Na^+^] losses (~45 mmol/L, 416 mg/h) were similar across sexes, aligning with previous research on team sports with large individual variations in sweat [Na^+^] [35]. However, males exhibited higher sweat [K^+^] concentrations, possibly reflecting increased intracellular fluid turnover [30], though the physiological implications of this difference are not yet fully understood. These findings underscore the importance of individualized hydration strategies, as SR and electrolyte losses vary between athletes [37]. While electrolyte supplementation protocols may be similar for both genders, fluid intake adjustments may be needed to account for the higher SR in males.

### 4.4. Limitations and practical applications

A small sample size can be considered a limitation in the present study; however, it represents the entirety of the lead country’s National Archery Team, comprising current Olympic, World, and European medalists. Thus, the findings provide valuable insights into the practices of some of the most elite archers globally. However, further studies should aim to include a larger and more diverse sample of archers from various teams and competitive levels to enhance the generalizability of the findings and provide a broader understanding of training and competition practices across different contexts. All the females tested were in the luteal phase of their menstrual cycle during this competition and we do not believe that responses would differ during another phase as fluid balance appears to be unaffected [38].

The findings of this study highlight the importance of periodized nutritional and tailored hydration strategies for elite archers during a multi-day competition. Male and female archers exhibited differences in energy and carbohydrate intake, suggesting that periodized nutrition plans, adjusted for body size and physical demands during competition are essential to ensure adequate fueling throughout the tournament. Coaches and practitioners should emphasize periodized carbohydrate intake, aligning it with daily competition loads (e.g. higher intake on high-volume shooting days), while ensuring adequate hydration, as both sexes maintained euhydration, with males requiring more fluids due to higher sweat rates. Future research should expand sample sizes and explore interventions to refine these strategies further, ensuring they are adaptable across diverse competition environments and athlete profiles.

## 5. Conclusion

The findings suggest that world-class archers effectively modulate their CHO intake during multi-day competitions, aligning intake with the specific demands of each day. This modulation highlights the need for individualized nutritional periodization guidance to ensure optimal performance throughout the tournament. The findings also emphasize the need for individualized hydration and electrolyte replacement strategies in elite archery. Male archers demonstrated a higher SR, necessitating greater fluid replacement, while Na^+^ and K^+^ losses were relatively similar between sexes. The results of this study contribute to a more comprehensive understanding of archers’ nutrition and hydration requirements. These could help guide education for coaches and sports staff regarding tailoring CHO intake and hydration in addition to providing practical procedures for SR and/or sweat testing.
